# The Emerging Role of Amino Acid PET in Neuro-Oncology

**DOI:** 10.3390/bioengineering5040104

**Published:** 2018-11-28

**Authors:** Amer M. Najjar, Jason M. Johnson, Dawid Schellingerhout

**Affiliations:** 1 Division of Pediatrics, The University of Texas M.D. Anderson Cancer Center, Houston, TX 77030, USA; 2 Department of Diagnostic Radiology—Neuro Imaging, The University of Texas M.D. Anderson Cancer Center, Houston, TX 77030, USA; jjohnson12@mdanderson.org (J.M.J.); dawid.schellingerhout@mdanderson.org (D.S.)

**Keywords:** magnetic resonance imaging, positron emission tomography, amino acid PET, central nervous system malignancy, pseudoprogression, pseudoresponse

## Abstract

Imaging plays a critical role in the management of the highly complex and widely diverse central nervous system (CNS) malignancies in providing an accurate diagnosis, treatment planning, response assessment, prognosis, and surveillance. Contrast-enhanced magnetic resonance imaging (MRI) is the primary modality for CNS disease management due to its high contrast resolution, reasonable spatial resolution, and relatively low cost and risk. However, defining tumor response to radiation treatment and chemotherapy by contrast-enhanced MRI is often difficult due to various factors that can influence contrast agent distribution and perfusion, such as edema, necrosis, vascular alterations, and inflammation, leading to pseudoprogression and pseudoresponse assessments. Amino acid positron emission tomography (PET) is emerging as the method of resolving such equivocal lesion interpretations. Amino acid radiotracers can more specifically differentiate true tumor boundaries from equivocal lesions based on their specific and active uptake by the highly metabolic cellular component of CNS tumors. These therapy-induced metabolic changes detected by amino acid PET facilitate early treatment response assessments. Integrating amino acid PET in the management of CNS malignancies to complement MRI will significantly improve early therapy response assessment, treatment planning, and clinical trial design.

## 1. Introduction

Malignancies of the central nervous system (CNS) account for an estimated 23,000 cases and over 16,000 deaths each year [1]. Cerebral gliomas are second to meningiomas in frequency and account for the highest number of cancer mortalities in adults under the age of 35 [2]. Brain tumors arising from metastasis originating from peripheral tumors such as lymphoma, melanoma, lung, and breast cancer occur at an even a higher rate with an incidence of 9–17% [3].

The most recent 2016 World Health Organization (WHO) Classification of Tumors of the Central Nervous System defines CNS malignancies within four categories (grades I, II, III, and IV) based on molecular parameters and histology [4]. Grades I and II gliomas are devoid of anaplastic features and are classified as low-grade gliomas (LGG). High-grade gliomas (HGG) are classified in grades III and IV and include the most aggressive form, glioblastoma (grade IV), which has a median overall survival of 1.5 years [5]. 

The complexity and diversity of CNS malignancies necessitate a multifaceted approach to therapy that includes surgery, radiation treatment, chemotherapy and, more recently, immunotherapy. Historically, therapy of CNS tumors entailed surgery and radiotherapy. Improving outcomes relied on radiotherapy dose escalation and responses were measured by overall survival. With the advent of chemotherapeutics and immunotherapy (bevacizumab), radiographic assessment became necessary to assess immediate responses manifested in anatomic and molecular changes. Thus, imaging became an integral component of every stage of CNS disease management providing information that is critical to staging, formulating preoperative strategies, monitoring therapy response, surveillance, and prognosis. 

## 2. Role of Vascularity in Magnetic Resonance Imaging of CNS Tumors

Magnetic resonance imaging (MRI) is the primary diagnostic method given its high soft tissue contrast, spatial resolution, low risk, ready availability and relatively low cost. Intensity contrast between tumor mass and surrounding brain tissue along with anatomical distortions of normal brain structures and contrast-enhanced regions typically delineate tumors in MR images although the tumor boundaries are often notoriously difficult to demonstrate accurately by imaging. T1- and T2-weighted and fluid-attenuated inversion recovery (FLAIR) are the standard sequences utilized. Compared to healthy brain tissue, CNS tumors typically appear hypointense to myelinated white matter on T1-weighted images and hyperintense on T2. Other structural characteristics associated with tumor mass may include cysts, necrosis, hemorrhage, and calcification.

CNS tumors are generally hyper-vascularized in contrast to the highly structured and selectively permeable blood-brain barrier (BBB), which acts to protect the privileged chemical environment of the brain. The BBB is formed by tight junctions between endothelial cells supported by pericytes and astrocytic foot processes limiting permeability to the vast majority of circulating agents [6]. Although microglia within the vicinity of blood vessels can repair a transient injury to the BBB [7], pathologies of the brain can compromise the integrity of the barrier increasing permeability to large therapeutic agents such as antibody drugs [8,9,10]. Access of anti-CTLA antibodies and bevacizumab, for example, depend explicitly on a compromise of the BBB [11].

In contrast to the highly integrated nature of the BBB [12,13], tumor vascularity is irregular, leaky, and poorly structured. This abnormality gives rise to permeability, interstitial fluid pressure, hypoxia, necrosis, and edema characteristically exhibited by glioblastomas. Aberrant tumor vascularity can be established within the structured BBB environment by metastatic cancer cells that are capable of breaching and penetrating the tight junctions of the barrier through adherence and proteolytic processes that mimic leukocyte extravasation. Once established beyond the BBB, the metastatic tumor microenvironment signals the development of a new heterogenic vascular supply characterized by increased permeability due to altered pericyte composition [14]. This leaky neovasculature can favorably influence the delivery of therapeutics by way of leakiness but may also unfavorably raise interstitial pressures to resist penetration of therapeutic agents [12].

The vascular disparity between the tumor and healthy brain tissue facilitates contrast-enhanced tumor resolution with gadolinium agents for critical response assessments of CNS tumors by MRI [15,16,17]. Increased tumor vascularity is a surrogate of elevated proliferation and aggressiveness and has been employed to delineate tumors through perfusion contrast-enhancing agents to diagnose and monitor brain tumor response. Congruently, the hypervascularity of glioblastomas has also been exploited as a therapeutic target of antiangiogenic drugs such as bevacizumab. Although bevacizumab has yielded little improvement of overall survival, an undefined subset of glioma patients do receive survival benefit from this agent, and a marginal improvement of progression-free survival and quality of life has justified the use of the drug in combination with standard-of-care regimens [18].

## 3. Limitations of Treatment Response Assessments by MRI

The role of imaging in CNS tumor management has evolved to meet the needs of advances in therapy. Early therapeutic approaches relied primarily on resection and postoperative radiotherapy of CNS malignancies. These measures provided a survival benefit which was reflected in overall survival as the primary endpoint. Renewed efforts over the last 30 years to improve therapeutic outcomes through radiation dose escalation and adjuvant chemotherapy necessitated formulating new early response assessment parameters to provide objective and mechanistic insights into treatment response [19]. Consequently, the Macdonald criteria were established in 1990 as a means of reporting early radiographic response based on contrast-enhanced computed tomography and MRI [20]. The Macdonald criteria use contrast enhancement metrics to objectively stratify therapeutic responses into four categories: (1) complete response, (2) partial response, (3) stable disease, and (4) progressive disease [20,21]. 

Progression-free survival is a more immediate assessment of therapeutic efficacy and serves the needs of a more precise readout for specific therapies. This requires an accurate imaging readout at chosen intermediate time points during therapy. However, the limitations of anatomic and volumetric measurements, compounded by the subjective interpretation of equivocal lesions have hindered the ability of current imaging modalities in providing an intermediate clinical readout. These inadequacies with the Macdonald metrics became apparent with recognition of contrast-enhancing or -diminishing artifacts elicited by radiation-induced necrosis and the alteration of vascularity by chemotherapy (temozolomide) and immunotherapies (bevacizumab) leading to misinterpretation of therapeutic responses. These artifacts have introduced new caveats in the interpretation of radiological data based on the Macdonald criteria. 

**Figure 1:**
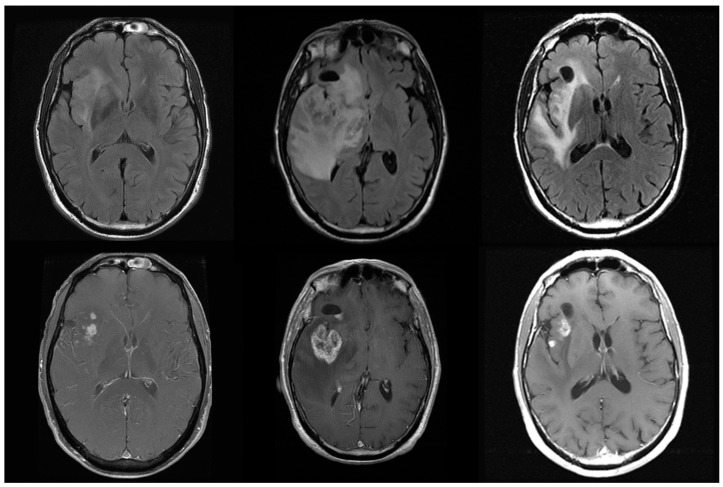
Glioblastoma (WHO IV) patient at presentation (left), shows an insular tumor with islands of enhancement. Following 60 Gy of radiation with Temozolamide 75 mg/m^2^ daily (middle column), there is an apparent increase in both enhancement and edema with mass effect. These worsened imaging findings resolve one month later with Decadron 6 mg twice daily, with a near return to imaging baseline. This apparent worsening on imaging is known as pseudoprogression and represents an inflammatory response to therapy that is difficult to distinguish from true progression. Pseudoprogression complicates the imaging and clinical management of glioma patients.

Therapies affecting vascular permeability and perfusion give rise to the phenomena of pseudoprogression and pseudoresponse where the tumor alternately appears worse or better on imaging due to spurious effects on the vasculature. This presents a formidable challenge to the accurate and objective evaluation of therapeutic outcomes, as intermediate time points gauging progression-free survival become very difficult to interpret (Figure 1).

Increased BBB permeability, necrosis, inflammation, and hemorrhage may be instigated by radiation-induced injury along with edema, and can appear mass-like on imaging. Unlike a tumor mass, however, these contrast-enhancing regions are not associated with cellular density or vascular intensity but represent a site of tissue breakdown and leakiness that mimics many of the imaging attributes of a tumor. The combination of radiotherapy and cytotoxic agents will often cause this effect and is known as pseudoprogression, which mimics the imaging appearance of tumor progression and can even cause clinical symptoms due to mass effect but is not due to true progressive disease. Checkpoint-blockade immunotherapy is also likely to present as pseudoprogression [22].

Conversely, antiangiogenic agents, such as bevacizumab, may cause short-term decreased perfusion and reduced contrast enhancement due to "normalization" of the tumor vasculature leading to a false appearance of treatment response, or pseudoresponse, not associated with a real anti-tumor effect or improved overall survival [23,24]. Bevacizumab’s effects can cause imaging to show decreased T1 contrast enhancement, edema, and mass effect. This imaging outcome, however, does not correlate with long-term benefit or improved overall survival [24].

The challenges and limitations associated with MRI limit objective assessment of timely therapy response assessment and prognostication. Contrast-enhanced MRI is mostly a function of BBB integrity and tumor vascularity and is, therefore, a nonspecific form of tumor mass characterization that is prone to equivocal interpretation. These imaging artifacts may be instigated by transient effects of therapeutic interventions enhancing contrast leading to overestimation of their therapeutic efficacy. Therefore, evaluation over multiple time points is critical to circumvent misinterpretation of the single-point transient anomaly.

## 4. Positron Emission Tomography of CNS Tumors

Positron emission tomography (PET) is an imaging modality that is based on the preferential uptake and retention of radiolabeled tracers by the target tissue. These radiotracers mimic or are sometimes chemically identical to, metabolites that are avidly taken up by proliferative cells to meet their energy or biomass demands. Tumor cells have a higher tendency to absorb these metabolites generating contrast in uptake between tumor mass and surrounding healthy tissue.

The development of PET radiotracers has addressed some of the limitations of structural MRI in discerning pseudoprogression from true progression. Tumor delineation based on metabolic radiotracer uptake offers a functional basis of detection that yields enhanced differentiation of tumor from equivocal lesions over MRI, improved delineation of tumor boundaries for surgery and radiotherapy planning, differentiation between tumor progression and treatment-related responses, and monitoring of tumor response to therapy.

Over the last four decades, PET with 2-deoxy-2-[^18^F]-fluoro-D-glucose (^18^F-FDG PET) combined with computed tomography has emerged as a standard-of-care imaging modality for the detection of tumors based on their elevated glucose metabolic rate (Warburg effect). The utility of ^18^F-FDG PET in imaging brain tumors, however, is hindered by the elevated background uptake level of glucose by normal brain tissue generating little discernable contrast. Moreover, immune cell activation within inflammatory responses also exhibits elevated glucose metabolism further obscuring distinction from brain or tumor tissue [25]. While metabolic contrast between tumor and healthy brain for FDG is low, there are considerable differences for amino acid and nucleotide metabolism. Tumor cell division (DNA replication) and growth (biomass generation) demand amino acid and nucleotide building blocks to meet the needs of the rapidly proliferating cells.

Accordingly, elevated DNA replication of tumor cells can be readily discerned using 3′-deoxy-3′-^18^F-fluorothymidine (^18^F-FLT) PET against the background of the relatively quiescent normal brain cells. Importantly, ^18^F-FLT does not readily accumulate in inflammatory lesions, as is the case with ^18^F-FDG, and is, therefore, able to report on cellular proliferation. Inflammatory immune cells primarily undergo aerobic glycolysis during activation, and are, therefore, not detected by ^18^F-FLT. The specificity of ^18^F-FLT for cellular proliferation facilitates its utility in reporting on tumor response to therapy [26] and has been shown to a better predictor of overall survival than MRI following treatment of recurrent gliomas with bevacizumab and irinotecan [27]. Moreover, ^18^F-FLT uptake detects treatment response early and is highly predictive of overall and progression-free survival following bevacizumab treatment [28,29]. However, the need for BBB breakdown for ^18^F-FLT limits its reliability in reporting on brain tumor treatment response [30].

### 4.1. Amino Acid PET

Amino acid PET has been evaluated extensively for detecting tumor mass based on metabolic tumor volume and is playing an increasingly important role in the management of CNS tumors. Ambiguous brain lesions that may complicate an accurate diagnosis of brain malignancies include hemorrhage, necrosis, edema, infarctions, abscesses, and inflammation. These extraneous lesions may be caused by a response to radio- and chemotherapy (inflammation and necrosis) or secondary anatomical disruptions caused by tumor outgrowth. In contrast to structural MRI, which cannot accurately differentiate lesion traits, amino acid radiotracer accumulation is a function of tumor avidity for the carbon source to meet its high demands for biomass and energy generation. This differential uptake of amino acid tracers can be exploited to specifically delineate cellular mass and tumor boundaries from surrounding normal tissue. Analogs of methionine, tyrosine, phenylalanine, alanine, and leucine have been evaluated for their specificity in delineating CNS tumors yielding nearly equally effective outcomes.

The ability of amino acid tracers to cross the BBB is a crucial advantage that transcends the limitations of contrast-enhanced MRI, which relies on leaky vascularity or compromise of the barrier for delivery of contrast enhancing agents. Compromise of the BBB by tumor growth does not appear to be a requirement for amino acid tracer permeability [31,32]. The intracellular uptake of amino acid tracers by the tumor cells is an active process facilitated by the L (large) transport system with subtypes LAT1 and LAT2 and is, therefore, more specific in reporting on live proliferating cells rather than structural changes [33,34,35,36]. This is further evidenced by dexamethasone or antiangiogenic treatment with bevacizumab not affecting amino acid uptake by brain tumors [37,38]. Prior or ongoing treatment with temozolomide, however, may impact the tumor-to-background ratio of amino acid uptake and must be considered in therapy response assessments [39].

The PET radiotracer [^11^C-methyl]-l-methionine (^11^C-MET) is the most well-established and utilized amino acid probe. ^11^C-MET PET has been shown to be useful in delineating ependymomas, medulloblastoma, and astrocytomas in pediatric patients and can also effectively differentiate between radiation-induced brain tissue injury and tumor recurrence [40,41,42,43]. It is superior to MRI in differentiating tumor necrosis following gamma knife radiosurgery from recurrent tumor [44] and offers accurate tumor size correlation of WHO grades II and III meningiomas [45].

**Figure 2:**
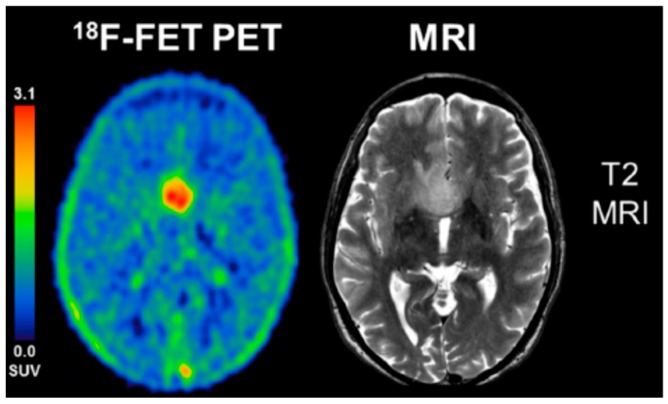
^18^F-FET PET summation image from 5–15 min is contrasted to a T2 MR image in this patient with a WHO III astrocytoma, IDH-wild-type, without 1p/19q co-deletion. This was a non-enhancing tumor on MRI. Note excellent tumor to background contrast for the FET PET image, with standard uptake values (SUV) of up to 3. Adapted from Unterrainer et al. *Eur. J. Nucl. Med. Mol. Imaging* 2018, 45, 1242 with permission [51].

The short 20-min half-life of the ^11^C, however, limits the practical implementation of ^11^C-MET PET to highly specialized imaging centers with onsite cyclotron facilities. Consequently, ^18^F-labeled amino acid tracers (with a half-life of 110 min), such as *O*-(2-^18^F-fluoroethyl)-l-tyrosine (^18^F-FET), 3,4-dihydroxy-6-^18^F-fluoro-l-phenylalanine (^18^F-FDOPA), and anti-1-amino-3-^18^F-fluorocyclobutane-1-carboxylic acid (^18^F-FACBC, fluciclovine, a non-natural amino acid) have been developed among many others to overcome this logistical limitation. Amino acid radiotracers, such as ^18^F-FET, also exhibit tumor type-specific kinetics that can facilitate differential diagnosis of CNS tumors [46,47,48,49,50,51] (Figure 2). Metabolic changes in response to bevacizumab treatment of glioblastoma identified by ^18^F-FET PET imaging occur earlier than morphologic changes providing a more immediate indication of tumor progression than changes detected by MRI [52]. Both ^11^C-MET and ^18^F-FACBC were able to differentiate tumor from equivocal lesions (edema) [53]. 

Although ^18^F-FDOPA exhibits differential tumor uptake, its similarity to dopamine results in preferential accumulation in the striatum presenting an obstacle to accurately demarcating gliomas involving this region of the brain [54]. In comparison to ^11^C-MET, ^18^F-FACBC yields lower background accumulation levels (i.e., higher tumor-to-brain signal ratios) and, hence, higher detection sensitivity and specificity. ^18^F-FDOPA PET metabolic tumor volume measurements at two weeks following antiangiogenic treatment are highly predictive of outcome. A first follow-up scan at two weeks predicted increased overall survival and progression-free survival. Responders identified based on ^18^F-FDOPA-PET survived 3.5 times longer in contrast to responders identified by MRI who lived 1.5 times longer. ^18^F-FDOPA PET is more accurate at identifying responders only two weeks following antiangiogenic treatment [55].

### 4.2. Response to Therapy

Specific detection of early responses to therapy is a significant advantage of amino acid PET as it can specifically differentiate the cellular component of a tumor mass from inflammatory and necrotic lesions. Amino acid PET detects metabolic changes, which occur earlier than morphological changes detected by MRI in response to chemotherapy, radiation treatment, or antiangiogenic treatment. Favorable outcomes following three cycles of temozolomide treatment can be predicted by reduced ^11^C-MET uptake in high-grade glioma patients [56,57]. Furthermore, the tumor-associated breakdown of the BBB does not appear to be a requirement for amino acid tracer permeability resulting in more accurate tumor boundary delineation even after vascular normalization by bevacizumab [31,32]. ^18^F-FET and ^18^F-FDOPA can be used to detect response to bevacizumab treatment before the appearance of morphological changes and thus provide a much earlier prediction of response [52,55,58]. In preclinical glioma models, ^18^F-FET PET reports on early response to combination therapy with temozolomide, interferon-beta, or bevacizumab [59]. Management of recurrent high-grade glioma patients treated with bevacizumab and radiotherapy using ^18^F-FET PET and MRI is cost effective and may potentially enhance the treatment quality. These outcomes are realized by the avoidance of unnecessary costs associated with overtreatment and unnecessary side effects [60].

### 4.3. Grade Differentiation

Differentiation between low and high-grade gliomas by imaging is a potentially beneficial diagnostic tool that would enhance the management of CNS tumors. Generally, WHO grade I and II tumors and a significant number of WHO grade III gliomas are contrast non-enhancing on MRI limiting tumor burden and therapy response assessments. Furthermore, differentiation between LGG and HGG by MRI remains challenging. 

The utility of amino acid PET to differentiate between LGG and HGG based on the level of radiotracer uptake has been assessed in a limited number of studies. Generally, amino acid uptake is higher in grade III/IV gliomas compared to grade I/II [61,62,63]. [N-methyl-^11^C] alpha-Methylaminoisobutyric acid (^11^C-MeAIB), an amino acid analog, can differentiate between low-grade and high-grade astrocytoma as determined by the tumor to normal brain uptake ratio [64]. Differential uptake of alpha-[^11^C] methyl-l-tryptophan (^11^C-AMT) can be used to identify LGGs, even if they are not contrast-enhancing on MRI, and distinguish them from HGGs based on tumor-to-cortex radiotracer uptake [65].

Despite differential LGG/HGG imaging in many studies, a recent systematic review of amino acid PET of LGGs has revealed a difficulty in interpretation due to inconsistencies in radiotracer uptake and different correlations between uptake ratios and LGG molecular status [66]. Radiotracer uptake intensities for each group can vary widely resulting in overlap and unreliable preoperative grading [67]. 

Kinetic PET imaging, however, may offer a better differentiating parameter based on the differing rate of radiotracer uptake by LGG and HGG. Time-activity curves of grade II tumors exhibit a slow, steady increase compared to the rapid uptake of amino acid radiotracers by grade III/IV tumors. Thus, LGG and HGG can be more reliably differentiated based on a dynamic or dual-time-point PET than static or endpoint imaging [47,48,61,68,69,70].

### 4.4. Prognostication

As an extension to its capacity to report on the early response to therapy, amino acid PET has proven to be a reliable prognostic tool in predicting overall and progression-free survival. Decreased ^18^F-FET uptake following radiochemotherapy was associated with overall survival compared to increased or stable uptake [71,72]. True responders to bevacizumab treatment following recurrent glioma identified by ^11^C-MET PET at eight weeks were predicted to have more favorable prognoses [73]. Determination of metabolic tumor volume of ^11^C-MET-PET is prognostic of progression-free survival in high-grade glioma patients [74] and predictive of patients developing recurrent malignant glioma [75].

Amino acid uptake may also serve as a reliable predictor of survival in patients with LGGs. ^18^F-FET PET combined with anatomic MRI predicted outcome and progression-free survival [76]. Kinetics of amino acid uptake have also been demonstrated to be useful in predicting regions of malignant transformation and progression within LGG [70,77]. Detection of metabolic abnormalities in LGGs by amino acid PET following treatment may also serve as a predictor of tumor recurrence [78].

### 4.5. Biopsy Guidance

Diagnostic inaccuracies based on image interpretation carry risk and compromise CNS tumor therapy outcomes. Biopsy provides diagnostic accuracy at a higher rate than neuroimaging and is essential to tumor grading and treatment planning. Therefore, image-guided stereotactic biopsy of tumor volumes is crucial to achieving higher diagnostic yields. Delineation of tumor extent and identification of regions of higher grade and density with ^18^F-FET and ^18^F-DOPA PET proved useful for biopsy guidance and resection planning in most cerebral glioma cases compared to ^18^F-FDG [67,79,80,81]. Needle guidance to hypermetabolic foci by PET produce a higher diagnostic yield than MRI reducing sampling to a single trajectory. Reducing sampling frequency through accurate image data is particularly important to minimize the risks associated with a biopsy of intrinsic infiltrative brainstem malignancies [82].

### 4.6. Case Studies Validating Amino Acid PET

Some case studies illustrate the advantages of amino acid PET over MRI in delineating tumor extent, informing on response to therapy, and detecting disease recurrence. Amino acid uptake has also been shown to be consistent with exhibited symptoms and biopsy results. A unique case study involving a 37-year old woman with a “butterfly” glioblastoma exemplifies tumor extent delineation by amino acid PET. MRI findings were equivocal and imprecise, indicating bifocal growth with slight contrast enhancement despite the large extent of the bilateral tumor mass. ^11^C-MET PET was more diagnostically precise than contrast-enhanced MRI in delineating a broad and continuous tumor mass [83].

Amino acid PET has also proved valuable in detecting recurrence and differentiating it from pseudoprogression as confirmed by biopsy. Detection of recurrence by PET was illustrated by high ^18^F-DOPA uptake in the left parietal lobe of a 46-year-old patient, where resection of a glioma had taken place two years earlier. ^18^F-FDG uptake was not elevated and did not indicate any abnormalities. Recurrence of the tumor in this patient was confirmed by biopsy demonstrating the accuracy of amino acid PET in detecting metabolic tumor volume [84]. Similarly, pseudoprogression has been reliably determined by amino acid PET in other case studies. Morphological changes on MRI suggested recurrent tumor eight months following radiochemotherapy in a glioblastoma patient. ^18^F-FET PET, however, was negative for focal uptake and, indeed, histopathology of the resected tumor revealed necrotic tissue consistent with pseudoprogression. The lesion regressed in follow-up MRI further confirming the diagnosis [85].

In contrast to MRI, consistency of amino acid uptake with patient symptoms has been demonstrated in a number of case studies. Vision problems exhibited by 16-year old female optic pathway glioma patient correlated with an increase in ^11^C-AMT uptake indicating recurrence. ^11^C-AMT uptake decreased upon chemotherapy and radiotherapy and correlated with a symptomatic improvement [86]. Likewise, ^18^F-MET PET was able to detect recurrence of a benign oligodendroglioma in a patient exhibiting recurrent temporal epileptic seizures 15 months following resection. ^18^F-MET PET uptake was high in the region of the previous tumor. Secondary resection and biopsy confirmed recurrence as predicted by PET [87]. In both of these cases, MRI did not reveal morphological changes indicating tumor recurrence and was inconsistent with exhibited patient symptoms.

## 5. Combined PET/MRI for the Management of CNS Tumors

The strength of MRI in providing high anatomical resolution of soft brain tissue coupled with the specificity of amino acid PET in delineating CNS tumors promote combining the modalities to improve diagnostic specificity in the management of CNS malignancies. When combined with MRI, ^11^C-MET-PET has been shown to be a reliable identifier of true responders to bevacizumab therapy with favorable prognoses [73,88,89]. Furthermore, improved diagnostic confidence of primary low-grade astrocytoma and high-grade astrocytomas [90] and reliable early differentiation between tumor recurrence and radionecrosis in high-grade glioma patients have been demonstrated [91]. The benefits of combined PET/MRI have also been extended to pediatric patient management and found to be well-tolerated and have been recommended for early prediction of response [92].

## 6. Conclusions and Future Perspectives

**Figure 3:**
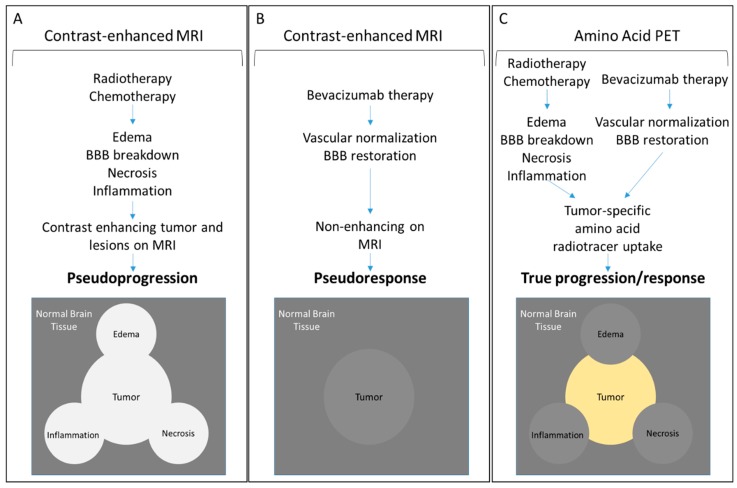
Schematic representation of MRI and PET outcomes following treatment of CNS tumors. (**A**) Radiotherapy and chemotherapy of CNS tumors can lead to edema, necrosis, inflammation, and breakdown of the BBB creating contrast-enhancing lesions that obscure tumor boundaries and lead to pseudoprogression. (**B**) Conversely, vascular normalization following bevacizumab treatment, without an associated anti-tumor response, can diminish contrast enhancement yielding a false appearance of treatment response or pseudoresponse. (**C**) Amino acid PET reports specifically on the accumulation of the radiotracers within the cellular component of the tumor mass reflecting correct tumor boundaries and immediate metabolic changes in response to therapy.

Metabolic changes detected by amino acid PET occur in response to therapy sooner than morphological and structural changes providing an earlier response to therapy assessment and prognosis. Moreover, metabolic uptake of radiolabeled amino acids is specific to the proliferating cellular component of the tumor, occurs independently of the status of the blood-brain barrier, and excludes non-cellular lesions created by necrosis, edema, and inflammation. As summarized in Figure 3, these advantages offered by amino acid PET translate into precise tumor boundary delineation and early treatment response assessments that transcend MRI-based limitations of pseudoprogression and pseudoresponse.

The advantages provided by amino acid PET justify its future implementation as a standard procedure in defining biopsy sites, planning surgical resection and radiotherapy, prognostication, and treatment response assessment [93,94]. These tangible benefits have been realized leading to the recommendation by the Response Assessment Neuro-Oncology (RANO) working group for the inclusion of amino acid PET in the management and diagnosis of brain tumors in conjunction with conventional MRI [95]. Complementing the limitations of MRI with the strengths of amino acid PET will lead to significant improvements in all aspects of CNS tumor management.
